# Research Progress in Microporous Materials for Selective Adsorption and Separation of Methane from Low-Grade Gas

**DOI:** 10.3390/molecules29184404

**Published:** 2024-09-16

**Authors:** Dongrui Su, Panpan Chen, Cunlei Li, Yongfei Yan, Ranlei Zhao, Qingyou Yue, Yupeng Qiao

**Affiliations:** 1 College of Petroleum Engineering, Liaoning Petrochemical University, Fushun 113001, China; 17614103725@163.com (D.S.);; 2 School of Mechanical Engineering, Liaoning Petrochemical University, Fushun 113001, China

**Keywords:** low-grade gas, CH_4_/N_2_, adsorption separation, microporous materials

## Abstract

Given that methane (CH_4_) and nitrogen (N_2_) have similar properties, achieving high-purity enrichment of CH_4_ from nitrogen-rich low-grade gas is extremely challenging and is of great significance for sustainable development in energy and the environment. This paper reviews the research progress on carbon-based materials, zeolites, and MOFs as adsorbent materials for CH_4_/N_2_ separation. It focuses on the relationship between the composition, pore size, surface chemistry of the adsorbents, CH_4_/N_2_ selectivity, and CH_4_ adsorption capacity. The paper also highlights that controlling pore size and atomic-scale composition and optimizing these features for the best match are key directions for the development of new adsorbents. Additionally, it points out that MOFs, which combine the advantages of carbon-based adsorbents and zeolites, are likely to become the most promising adsorbent materials for efficient CH_4_/N_2_ separation.

## 1. Introduction

Currently, energy resource shortages, environmental pollution, and the intensification of the greenhouse effect are major problems and challenges facing humanity. With the proposal of carbon neutrality and carbon peaking goals, the task of carbon emission reduction has become more urgent [1]. Unconventional natural gas, specifically coalbed methane (CBM), which mainly comprises CH_4_, is considered a relatively clean fossil fuel as its complete combustion only produces CO_2_ and H_2_O. It also serves as an important modern industrial raw material [2]. CH_4_ is listed as a greenhouse gas in the Kyoto Protocol. It is noteworthy that its ozone-depleting potential is six times that of CO_2_, and its contribution to the greenhouse effect is 21 times greater, making it a significant driver of the greenhouse effect [3].

With the acceleration of the global development of green and low-carbon energy, the consumption and demand for natural gas continue to increase, signaling the advent of the global natural gas era [4]. It is well known that as the transition from fossil energy to clean energy progresses, conventional natural gas resources can no longer meet the sharply increasing demand for natural gas [5]. Unconventional natural gas will become a strong guarantee for natural gas supply, with unconventional hydrocarbon resources gradually becoming the primary source of energy consumption, with coalbed methane playing a crucial role [6]. In fact, in recent years, approximately 40 billion cubic meters of low-concentration coal mine gas (CH_4_ ≤ 30%) have been directly vented, resulting not only in resource wastage but also diverging significantly from the post-2020 emission reduction targets set by the United Nations Framework Convention on Climate Change (UNFCCC) at the 21st Conference of the Parties (COP21) held in Paris.

Clearly, CH_4_ has multiple attributes; it is both an indispensable clean energy source for sustainable development and a carrier of environmental pollution and enhanced greenhouse effects [7]. Therefore, developing CH_4_ enrichment technologies to utilize CH_4_ from low-concentration gas is of great significance for maintaining atmospheric balance, mitigating global greenhouse effects, and reducing resource waste [8]. Efficient CH_4_/N_2_ separation is a critical step in the enrichment and recovery of low-concentration gas. This paper systematically elucidates the process methods for the adsorption separation of CH_4_/N_2_ and the latest advancements in microporous materials used for the adsorption separation of low-concentration gas. It also provides an outlook on future developments, aiming to offer insights into the development of microporous materials for CH_4_/N_2_ adsorption separation.

## 2. Adsorption Separation Technology and Processes

The separation of N_2_ is a crucial step and technical challenge in the purification and refinement of low-grade gas. Due to the highly similar physicochemical properties of CH_4_ and N_2_, as shown in Table 1, CH_4_ and N_2_ are typical nonpolar molecules with no dipole moments. Except for the critical temperature, CH_4_ and N_2_ have similar kinetic diameters, polarizabilities, and quadrupole moments, with only a certain difference in boiling points, making their effective separation particularly difficult [9,10,11,12,13,14].

Typical purification processes for low-concentration CH_4_ include the removal of light hydrocarbons (C_2_^+^), dehydration, decarbonization, desulfurization, demercurization, and liquefaction-based nitrogen removal, with the latter being the most energy-intensive step in CH_4_ purification, severely limiting the large-scale application of low-concentration CH_4_ resources. To date, the purification of low-grade gas mainly employs techniques such as cryogenic distillation [16], membrane separation [17], chemical absorption [18], hydrate technology [19], and adsorption separation [20,21]. Table 2 systematically summarizes the principles and technical advantages and disadvantages of low-grade gas purification methods.

Among the various technologies for enriching low-concentration methane, adsorption separation stands out as the most promising candidate for industrial application. Adsorption is generally classified into two types: physical adsorption, also known as Van der Waals adsorption, and chemical adsorption. Physical adsorption occurs as a result of the mutual attraction between the adsorbent and adsorbate, leading to their adherence without the formation of chemical bonds. This process is reversible, allowing the adsorbate to be desorbed from the adsorbent surface under varying conditions. Chemical adsorption, on the other hand, involves the creation or breakage of chemical bonds between the adsorbent and adsorbate through chemical interactions. It results in the formation of new, stronger bonds between the two, often making the process irreversible. Pressure swing adsorption (PSA) exploits the differences in adsorption capacities of various gas molecules by the adsorbent under varying pressures. At elevated pressures, the adsorbent exhibits a strong affinity for certain components (typically impurities) within the gas mixture, whereas at lower pressures, its adsorptive capacity towards these components diminishes. By periodically altering the operating pressure, an alternating process of adsorption and desorption can be achieved, facilitating the separation of gases. The primary models underpinning PSA encompass adsorption equilibrium models, mass transfer kinetics models, mass conservation models, energy conservation models, and momentum conservation principles. To streamline the analysis, several assumptions are typically made, such as the gases adhere to the ideal gas law, radial concentration and temperature gradients are neglected, axial diffusion and axial heat conduction are disregarded, and heat transfer and heat accumulation along the adsorbent bed wall are ignored. Since Skarstrom designed the first pressure swing adsorption (PSA) process, researchers have conducted extensive studies and optimizations. Today, pressure swing adsorption separation processes mainly include steps such as pressurization adsorption, pressure equalization (up/down), purging, and depressurization desorption to improve CH_4_ enrichment efficiency [22].

**Figure 1:**
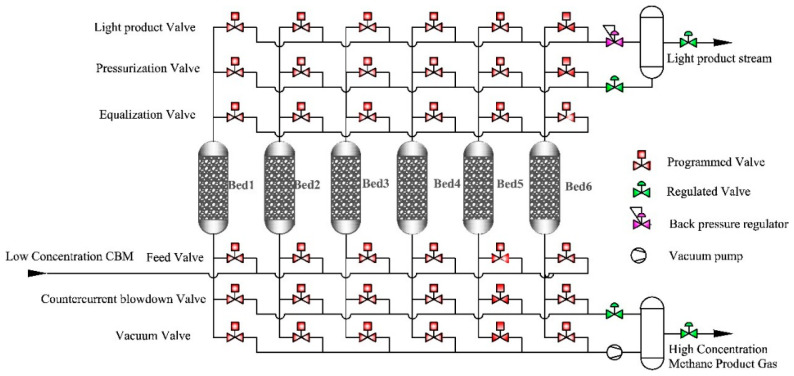
The schematic diagram of the six-bed VPSA process [24].

Zhang et al. evaluated the enrichment performance of ultra-low concentration gas (CH_4_ < 5%) using a carbon molecular sieve (CMS-3 KT) as an adsorbent and adopting micro-positive pressure vacuum pressure swing adsorption (~120 kPa) for CH_4_ enrichment from CH_4_/O_2_/N_2_ mixed gases. At an inlet flow rate of 200 mL/min, optimal enrichment performance was achieved within 0–3 min. The CH_4_ concentration in the collected product gas was nearly 2.5 times that of the feed gas, with a recovery rate of approximately 80%, and the O_2_ concentration reduced to about 3%. This process is of great significance for the removal of oxygen and the enrichment of CH_4_ from oxygen-containing low-concentration coalbed methane [23]. Lu et al. conducted research on the process of enriching low-concentration gas using activated carbon. They determined and calculated the adsorption thermodynamics and kinetics of CH_4_ and N_2_ on AC adsorbents through static volumetric isotherm measurement and breakthrough experiments. A mathematical model of the adsorption bed was also established, and a two-stage VPSA process using AC adsorbents for enriching low-concentration CH_4_ was developed, as shown in Figure 1. This process enriched and purified low-concentration CH_4_ with an initial concentration of 20% to over 90%, with a CH_4_ recovery rate of over 98% and an energy consumption of less than 0.504 kW·h·(m^3^ CH_4_)^−1^ [24].

**Figure 2:**
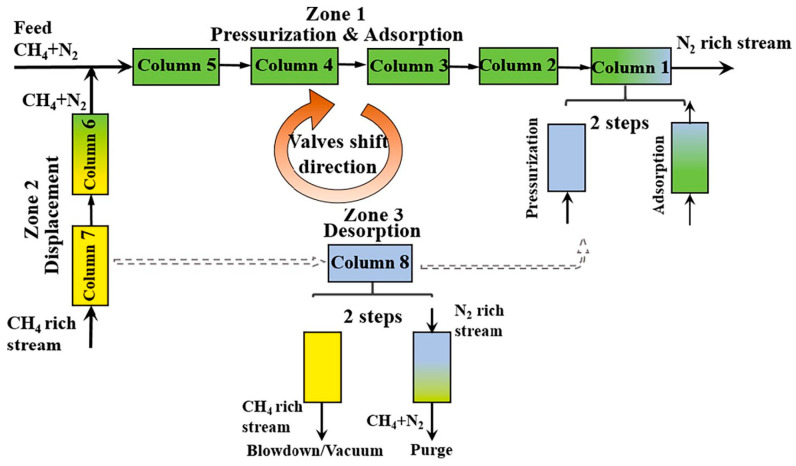
Schematic flow diagram of the eight-column VPSA process with SMB mode. Different colors represent changes in different state parameters (pressure, gas composition, etc.), and dotted lines represent the transition of the three areas [26].

To address the limitations of gas mixing at the feed inlet and the discontinuity in the axial concentration distribution in the adsorption column, Guo et al. introduced multiple feed inlets for enrichment and proposed a new dynamic feed dual reflux pressure swing adsorption process (DF-DR-PSA) for enriching low-grade CH_4_ (2.4 mol.%) and N_2_ mixtures. They conducted numerical simulations using Norit RB3 activated carbon adsorbent to compare the performance of the traditional DR-PSA process and the dynamic feed DF-DR-PSA process. Under the same energy consumption conditions, the DF-DR-PSA process showed improvements in both CH_4_ purity (53.5% vs. 47.5%) and recovery rate (81.1% vs. 72.2%) compared to the traditional DR-PSA process, solving the gas mixing issue at the feed inlet and enhancing CH_4_/N_2_ separation performance [25]. When the CH_4_ content in the feed gas is less than 20%, the proportion of recycled gas in the product gas and feed gas may be high, potentially reducing the performance of the VPSA process and increasing the energy consumption for recycling gas transport. To address this issue, Qian et al. proposed an improved integrated VPSA process operating in a simulated moving bed (SMB) mode, using commercial coconut shell activated carbon (AC) as the adsorbent. They applied an eight-column system to enrich CH_4_ from feed gas with 10–50% CH_4_ content, as shown in Figure 2, and compared the results of the improved VPSA process with traditional adsorption processes. The results demonstrated that the integrated VPSA process could successfully enrich and purify feed gas with 10% CH_4_ to produce 99.0% CH_4_ product gas, even using commercial activated carbon as the adsorbent [26].

Currently, there are many scientific issues that need to be addressed in the field of adsorbent materials and adsorption separation processes. Among these, the development and preparation of microporous materials for the efficient separation of CH_4_/N_2_ is a particularly prominent problem. Achieving effective control over the pore channels and surface structure of adsorbent materials at the nanoscale and designing high-performance adsorbent materials with multiple coupled separation principles will be an effective solution to overcoming the bottleneck in CH_4_/N_2_ efficient adsorption separation technology.

## 3. Porous Materials for CH_4_/N_2_ Adsorption Separation

CH_4_/N_2_ adsorption separation is primarily based on the differences in adsorption selectivity between the two gases on solid microporous materials. This selectivity arises from three mechanisms: equilibrium effects, kinetic effects, and steric effects [27]. Taking the equilibrium effect as an example, there are significant differences in the models used to describe the adsorption behavior of different adsorbents under different conditions. Commonly used models to describe adsorption behavior include the Langmuir model, the Freundlich model, and the BET model. The essence of adsorption separation lies in the development of high-performance adsorbents and the design of processes that match these materials. High-performance microporous adsorbent materials are crucial for adsorption separation because they significantly influence the mass transfer process and system efficiency. Excellent adsorption selectivity and adsorption capacity are fundamental requirements for superior adsorbents, along with other factors such as physicochemical stability, regenerability, and environmental friendliness. Currently, the porous materials used for low-concentration gas adsorption separation mainly include carbon-based materials, zeolite molecular sieves, and metal/organic frameworks (MOFs) [28].

### 3.1. Carbon-Based Adsorbent Materials

Carbon-based materials are widely used in the field of adsorption separation due to their advantages of simple preparation and readily available raw materials. Among these, activated carbon and carbon molecular sieves have good CH_4_/N_2_ separation coefficients and CH_4_ adsorption capacities, making them common carbon-based materials for adsorption separation [29,30,31]. The adsorption performance of some of these carbon-based materials is shown in Table 3.

**Figure 3:**
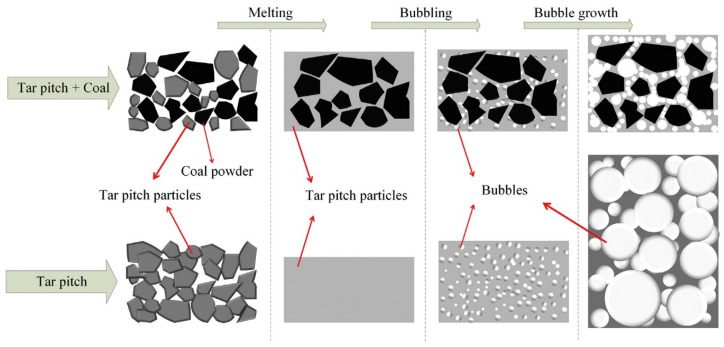
Illustration of a low-pressure foaming process by bubble growth in tar pitch with and without coal particles as additives [33].

Researchers have conducted extensive studies on the differences in adsorption performance due to various raw materials used in preparation. In 2015, Gu et al. prepared GAC (C-12) from anthracite using a pre-oxidation/carbonization/steam activation process, achieving a specific surface area of 412.51 m^2^/g with dominant pore sizes ranging from 5.0 nm to 6.43 nm. The CH_4_ adsorption capacity was 2.3 mmol/g, and the CH_4_/N_2_ selectivity was 3.17 (298 K, 1 MPa) [32]. Adding coal particles to asphalt has also been considered an effective method. In 2016, Arami-Niya et al. prepared AC using petroleum pitch and coal powder as raw materials through a low-pressure foaming method. Figure 3 illustrates the low-pressure foaming process of bubble growth in tar pitch with and without coal powder as an additive. This research group optimized the stability of asphalt foaming and product properties by controlling the ratio of KOH to coal pitch. They produced KOH0.5TP50 with a hierarchical pore structure, an open pore width of approximately 2 mm, well-developed micropores, a BET-specific surface area of 1044 m^2^/g, and an apparent density of 0.42 g/cm^3^ [33].

Subsequently, Yuan et al. used low-rank bituminous coal to prepare activated carbon C-12 with a high specific surface area of 3020 m^2^/g. Under experimental conditions of 6 MPa, its methane adsorption capacity was 13 mmol/g, and the CH_4_/N_2_ selectivity was 4.8 [46]. Additionally, coconut shells are often used for the preparation of activated carbon. Qu et al. conducted research on carbon materials prepared from coconut shells and found that the pore structure of carbonized coconut shells was poor. However, after KOH activation, the carbon materials had uniformly distributed regular micropores, higher specific surface area, and reduced polar functional groups on the surface, which facilitated the adsorption of weakly polar CH_4_ [47]. KOH is a strongly alkaline substance with significant corrosiveness and toxicity, and the preparation and use of carbon materials containing KOH may generate harmful wastewater and waste gases. These two approaches eschewed the highly polluting KOH activator, presenting an economical and green preparation method with broad application prospects for low-cost porous carbon materials. Tang et al. used rice as a carbon source to prepare granular carbon precursors through carbonization, followed by CO_2_ activation to produce rice-based carbon materials (PRCs) with narrow particle size distribution. Notably, the preparation process of granular PRC avoided the use of binders and KOH, reducing the usage of chemical reagents. The resulting PRC-850 had a BET-specific surface area of 776 m^2^/g. At 298 K and 100 kPa, its CH_4_ adsorption capacity and CH_4_/N_2_ selectivity reached 1.12 mmol/g and 5.7, respectively [48]. H_3_PO_4_-mechanical activation can significantly increase the adsorption volume of activated carbon. Pan et al. used bamboo sawdust as raw material and prepared samples using H_3_PO_4_-mechanical activation, with AC-1-400 achieving the largest pore volume (0.8 cm^3^/g) and the highest specific surface area (1966 m^2^/g). At 293 K, its CH_4_ adsorption capacity was 0.87 mmol/g, and the CH_4_/N_2_ selectivity was 3.38 [34].

**Figure 4:**
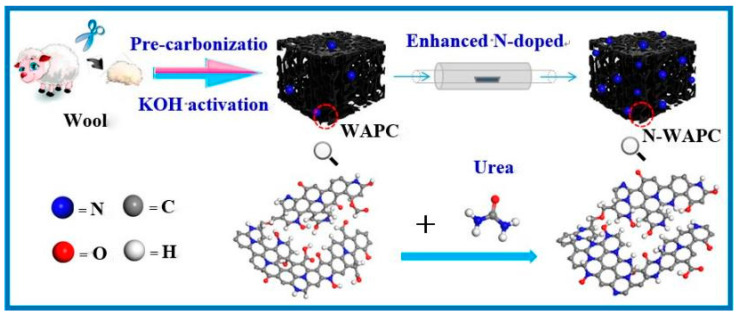
Schematic illustration of the synthesis of enhanced N-doped porous carbon [36].

Elemental doping has been proven to be an effective approach for modifying the pore structure of activated carbon, enhancing surface properties (including the specific surface area and micropore volume of the adsorbent material), optimizing the polarity of the adsorbent, and, consequently, improving its adsorption selectivity towards CH_4_/N_2_. This methodology offers a viable solution for optimizing the performance of adsorbent materials in targeted applications. In 2017, Yao et al. prepared a pyrolyzed fully Cl-substituted porous covalent triazine framework ClCTF-1-650 (at 650 °C), which exhibited an ultramicropore content of 98% and an N content of 12 at%. Due to its narrow pore size distribution, the N-doped porous carbon material significantly enhanced CH_4_ adsorption through weak interactions. At 298 K and 1 bar, the CH_4_ adsorption capacity was as high as 1.47 mmol/g, with a selectivity of 8.1, indicating that high nitrogen doping is an effective means of improving the selective adsorption of methane by microporous activated carbon [35]. Li activated waste wool with KOH and modified it with urea. The urea reacted with O functional groups, resulting in N-enhanced porous N-WAPC. The synthesis schematic is shown in Figure 4. Under experimental conditions of 298 K and 1 bar, the N-WAPC had a selectivity of 7.62 for equimolar CH_4_/N_2_ and a CH_4_ adsorption capacity of 1.01 mmol/g. N-WAPC shows great potential for efficient upgrading of CH_4_ from mixed gases [36].

Zhang et al. developed a porous carbon material featuring a large surface area of 774.0 m^2^/g, a high N-doping level of 4.81 atomic percent (at%), and a pore volume of 0.32 cm^3^/g through a low-temperature one-pot method (involving low-temperature activation and N-doping simultaneously). Experimental results indicate that the utilization of NaNH_2_ as an activator and the introduction of the N element effectively optimized the pore structure, promoting the formation of abundant porous structures with a significant number of hierarchical pores. This led to an increase in both the specific surface area and the micropore volume. Ultimately, by optimizing the pore-forming agent/carbon ratio and activation temperature, the prepared N-rich microporous carbon (OTSS-2-450) exhibited exceptional CH_4_/N_2_ selectivity of 4.9 at 298 K and 1 bar [37]. Wang et al. studied a method for preparing a series of porous carbon microspheres using (polyphosphazene-co-4,4′-sulfonyldiphenol) (PZS) as the raw material and carbonizing it at different temperatures. Among these, PZS-900 (carbonized at 900 °C) demonstrated excellent CH_4_ capture performance, with a specific surface area of 895.7 m^2^/g. The pore volume (0.3592 cm^3^/g) accounted for 76% of the total pore volume (0.4643 cm^3^/g), significantly enhancing CH_4_ adsorption performance [49]. Additionally, the hydrophilicity of carbon materials helps improve CH_4_/N_2_ separation performance. However, the preparation of N-doped microporous carbon typically requires an excess of corrosive KOH as an activator, limiting its industrial application. Since the process often uses corrosive KOH, Zhang et al. proposed a corrosion-free method to prepare N-doped microporous carbon materials by using potassium citrate as a non-corrosive activator and urea for N-doping. By adjusting the ratio of urea-to-potassium citrate, activation temperature, and time to modify the carbon materials, they found that ACK_2_N_1_ exhibited the best adsorption performance. The potassium citrate/urea ratio of 2:1 favored the generation of micropore volumes with pore sizes less than 1 nm and 1–2 nm. The microporous structure, especially ultramicropores (pore size < 1 nm), is beneficial for the adsorption of small molecules such as CH_4_. Properly increasing the amount of potassium citrate promotes the generation of a narrow spectrum, enhancing CH_4_ enrichment [38].

**Figure 5:**
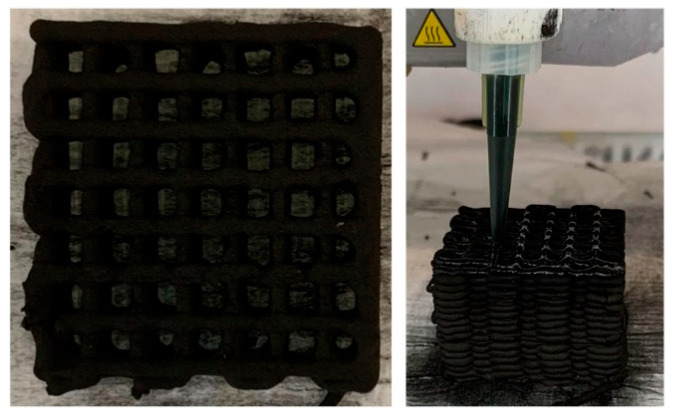
3D-printed activated carbon monolith [51].

The adsorption capacity and selectivity of activated carbon mainly depend on the particle size distribution and surface properties of the activated carbon. Li et al. modified coconut shell-based activated carbon with ammonia water and KOH. They compared the CH_4_/N_2_ separation performance of the modified and unmodified coconut shell-based activated carbon through equilibrium and dynamic adsorption experiments. The results showed that after modification with ammonia water, under optimal conditions of 12 h impregnation time and 10% modifier volume concentration, AC NH_3_·H_2_O-10%, 12 h at 298 K and 100 kPa had a CH_4_ adsorption capacity of 1.1 mmol/g and a selectivity of 4.62. This could be due to the introduction of amine and amide groups, which differentiate the adsorption energies of CH_4_ and N_2_ on AC. For the ammonia-modified samples, the introduced amine and amide groups greatly favored the selective adsorption of CH_4_ by differentiating the adsorption of CH_4_ and N_2_ on activated carbon. After KOH modification, the reduction of amine and hydroxyl groups weakened the CH_4_/N_2_ separation ability. The CH_4_ adsorption process mainly occurs on the surface of activated carbon, and its adsorption capacity is basically consistent with the volume of activated carbon. This is because of the capacity of surface basic groups; the larger the volume of AC, the more basic group capacity, thus, the stronger the adsorption performance. Increasing the capacity of basic groups through modified activated carbon may have great research value for enhancing CH_4_ adsorption performance [39]. Pan et al. conducted pressure swing adsorption experiments on low-concentration gas using a series of activated carbons. The activated carbon KCl/AC adsorbent treated with HCl had the largest specific surface area (1865 m^2^/g) and, at 298 K and 1 bar, the maximum CH_4_ adsorption capacity (7.89 mL/g), which was a 38.9% increase compared to the original AC (5.68 mL/g). The CH_4_/N_2_ selectivity of KCl/AC was 5.33, a 38.4% improvement over AC’s 3.85 [40]. Song et al. studied the chemical activation with phosphoric acid and KOH solutions and the high-temperature treatment in N_2_ or steam to prepare AC. Among them, AC-PS 800 treated with steam showed a significant increase in adsorption capacity due to the significant increase in micropore area, with pore sizes of 0.45–0.65 nm. Steam treatment of AC-P at 800 °C significantly increased the CH_4_ adsorption capacity to 6.5 mg/g, about twice that of the commercial sample GH-8 (3.2 mg/g). This confirmed that the combination of acidic chemical activation and high-temperature (i.e., 800 °C) steam treatment is a practical and effective method for enhancing CH_4_ adsorption performance [50]. Du et al. prepared a novel starch-based ultramicroporous carbon (SC) via an in situ ion activation method. These SCs were derived from starch and 1–6 wt.% acrylic acid, and the resulting materials were suitable for surface cation exchange. After activation, these SCs contained ultramicropores with a narrow pore size distribution of <0.7 nm, with SC-6 having an ultramicropore volume of 0.25 cm^3^/g, accounting for 78% of the total volume. At 100 kPa and 298 K, the CH_4_ adsorption capacity reached 1.86 mmol/g [41]. Chen et al. controllably prepared carbon adsorbents with uniform pore width from poly(vinylidene chloride) resin through activation-free pyrolysis. The microporous carbon adsorbents prepared by the activation-free pyrolysis method had a good specific surface area (>1100 m^2^/g) and a large specific pore volume (>0.37 cm^3^/g). At room temperature, the CH_4_ adsorption capacity was as high as 1.57 mmol/g, exceeding most of the best-performing adsorbents reported to date. Moreover, the optimal microporous carbon C-PVDC 700 showed the highest IAST selectivity for CH_4_ over N_2_ at 298 K and 100 kPa, with a value of 14.7. The material had numerous micropores distributed between 0.7 and 1.3 nm. Compared to traditional AC, the microporous carbon adsorbent obtained through pyrolysis had a higher proportion of mesopores and macropores. PVDC resin contains many oxygen-containing functional groups and numerous Cl-C-Cl functional groups, which are generally considered beneficial for CH_4_ adsorption. High selectivity and stability ensure that this material is an ideal adsorbent for LQNG upgrading [42]. Recently, Pereira et al. 3D-printed an integrated structure of Maxsorb-activated carbon using the polymer binder carboxymethyl cellulose (CMC). Based on the pre-established structure, the ink containing the adsorbent and binder was directly written in a layered manner. Using the ExDSL model and considering the equimolar mixture of CH_4_ and N_2_ at 303 K and 1 bar, the CH_4_ adsorption capacity was 1 mol/kg, with a CH_4_/N_2_ selectivity of 2.90. The novelty of this study lies in combining 3D printing technology with activated carbon adsorbents, as shown in Figure 5. The potential for CH_4_ and N_2_ separation using the Maxsorb-structured activated carbon adsorbent through direct ink writing (DIW) has been demonstrated [51]. Advanced 3D printing technology—direct ink writing (DIW)—is likely to become a valuable method for developing structured adsorbents at the laboratory scale and widely applied in industrial-scale promotion in the future.

Compared to activated carbon (AC), carbon molecular sieves (CMS) have uniform pore sizes, which utilize the differences in the kinetic molecular diameters of CH_4_ and N_2_, leading to different diffusion rates to achieve the separation of mixed gases [52]. The CH_4_/N_2_ separation process of carbon molecular sieves mainly includes two mechanisms: kinetic separation and adsorption equilibrium [53]. When the pore diameter of CMS is between the kinetic diameters of the gas molecules, the kinetic separation effect predominates.

**Figure 6:**
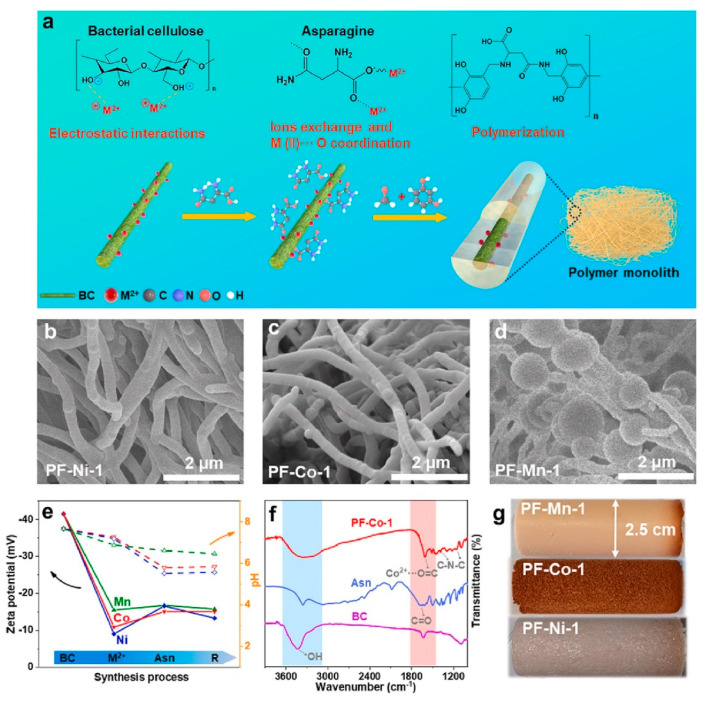
(**a**) Schematic of the synthesis strategy for PFs. (**b**–**d**) SEM image of PF-Ni-1, PF-Co-1, and PF-Mn-1. (**e**) The recording of Zeta potential and pH changes during the synthesis process. (**f**) FT-IR spectra of PF-Co-1, Asn, and BC. (**g**) Photograph of the as-obtained polymeric aerogels [45].

In 2004, Youn-Sang Bae studied the adsorption behavior of CH_4_ and N_2_ on CMS using volumetric methods and verified the feasibility of separating CH_4_/N_2_ mixtures on CMS through kinetic effects [54]. Subsequently, Olga Gorska et al. improved the pore size (mostly 0.8 nm) and specific surface area (1263 ± 50 m^2^/g) of the carbon molecular sieve DSV61Zn (CMS), which was prepared using “green” resources from willow and ZnCl_2_ as an activator. The selectivity reached up to 10.2 [55]. Zhang et al. prepared CMS using benzene as the deposition agent through chemical vapor deposition. The average width calculated by the DR equation was 0.47 nm. The maximum adsorption capacity was 1.41 mmol/g, and the selectivity reached up to 4.74, making it suitable for CH_4_/N_2_ adsorption separation [43]. Xiong et al. studied the adsorption equilibrium and kinetics of CH_4_, N_2_, and O_2_ on three types of carbon molecular sieves, as well as the corresponding separation performance. The experimental results showed that O_2_ had the largest diffusion time constant among the three gases, indicating the advantage of kinetic separation based on CMS in CMM deoxygenation and the key role of kinetic separation in CH_4_ enrichment [56]. Yang et al. modified coal-based carbon molecular sieves (CMS) using hydrocarbon-affinitive organic reagents such as docosane (C24), sodium dodecyl sulfate (SDS), and polyethyleneimine (PEI), followed by low-temperature plasma treatment. The best modification effect was observed for CMS-P-N, with a CH_4_ saturation adsorption capacity of 6.76 mmol/g and CH_4_/N_2_ selectivity of 3.32, indicating that low-temperature plasma treatment could provide a promising method for CH_4_/N_2_ adsorption separation [44]. Recently, Fu et al. prepared CMS with different oxidation degrees by combining carbon molecular sieves with KMnO_4_ oxidation. They studied the effects of microwave irradiation on the pores, functional groups, and high-pressure CH_4_ adsorption characteristics of the model substances. The results showed that microwave irradiation caused the reorganization of oxygen-containing functional groups in the carbon molecular sieves, blocking the micropores with diameters of 0.40–0.60 nm; meanwhile, the naphthalene and phenanthrene produced by the pyrolysis of macromolecular structures blocked the micropores of 0.70–0.90 nm in the carbon molecular sieves. These structural changes reduced the saturated CH_4_ adsorption capacity of the oxidized carbon molecular sieves by 2.91–23.28%, indicating that microwave irradiation could promote CH_4_ desorption. Additionally, the increase in mesopores in the oxidized carbon molecular sieves after microwave irradiation facilitated CH_4_ diffusion. Microwave irradiation is considered a highly promising technology for improving low-concentration methane enrichment [57]. Compared to AC and CMS, Li et al. reported a simple in situ growth method for preparing carbon nanofibers, as shown in Figure 6, and combined them with an ultramicroporous gas-selective layer for CH_4_/N_2_ separation. The thickness of the gas-selective layer could be precisely adjusted within the range of 90–525 nm. The optimal carbon nanofibers exhibited a high CH_4_/N_2_ selectivity of 6.8 and a CH_4_ adsorption capacity of 0.97 mmol/g, with CH_4_ diffusion kinetics two orders of magnitude faster than commercial activated carbon adsorbents. The high accessibility of adsorption sites and short diffusion paths of the nanofibers facilitated rapid mass transfer and enhanced the dynamic separation of CH_4_/N_2_. This study provided a novel approach for designing high-performance CH_4_/N_2_ adsorbents through a rational combination of abundant ultramicropores and short diffusion paths [45].

### 3.2. Zeolite Molecular Sieves

Zeolite molecular sieves are aluminosilicate crystals with a regular pore structure and excellent adsorption performance. Their basic structure consists of silicon/oxygen and aluminum/oxygen tetrahedra, which are interconnected by oxygen bridges, forming polyhedral cage structures with three-dimensional spaces. They have uniform pore size distribution and can sieve different molecules based on size [58,59]. In the separation of CH_4_/N_2_, zeolite molecular sieves can achieve efficient adsorption and separation of CH_4_ due to their unique pore size and adsorption characteristics. Additionally, synthesizing zeolite molecular sieves with specific pore sizes and structures can further improve the efficiency and selectivity of CH_4_/N_2_ separation [60]. Researchers have conducted extensive studies on this. Initial studies on the adsorption performance of conventional zeolite molecular sieves such as 4A, 5A, mordenite, and 13X for CH_4_/N_2_ showed that the difference in their equilibrium adsorption capacities was small, making it difficult to achieve efficient CH_4_/N_2_ separation [61,62,63,64]. Subsequently, methods such as ion exchange, changing the Si/Al ratio, and composite modification were used to further improve the CH_4_/N_2_ separation effect [65]. The adsorption performance of some of these zeolite molecular sieves is shown in Table 4.

Controlling the pore size of molecular sieves is an effective method for achieving the kinetic separation of CH_4_/N_2_ mixtures on molecular sieves. To address the issue of water content in low-concentration gas, especially when the adsorbent is a hydrophilic material, hydrophobic adsorbents are used to avoid destructive water absorption. Yang et al. developed three hydrophobic microporous high-silica zeolites: DDR (with 8-membered rings), silicalite-1 (with 10-membered rings), and beta (with 12-membered rings). The Si/Al ratios were 230, 1350, and 35, respectively. Experimental results showed that silicalite-1, with the most suitable pores for CH_4_ adsorption and the highest CH_4_/N_2_ selectivity (3.50), was more suitable for low-concentration CH_4_ enrichment compared to commercially used adsorbents zeolite 5A (2.5) and 13X (1.3) [66]. Kuznicki et al. adjusted the framework of silicate ETS-4 by high-temperature dehydration to modify the effective pore size, allowing gas molecules to enter the crystal interior. This so-called “molecular gate” effect can be used to customize molecular sieves with different adsorption properties, suitable for separating commercially important gas mixtures [73]. B. Majumdar et al. further studied the CH_4_/N_2_ adsorption of ETS-4 ion-exchange variants based on the principle that the 8MR channel contracts as the dehydration temperature increases (at the molecular scale). They found that at lower dehydration temperatures, CH_4_ adsorbed more strongly and diffused more slowly than N_2_. Gradual dehydration reduced equilibrium capacity and diffusion rates for both gases, affecting the larger CH_4_ molecules more than the relatively smaller N_2_ molecules, increasing diffusion coefficients and reducing equilibrium selectivity. Eventually, equilibrium selectivity reversed, leading to a maximum kinetic selectivity value of 205 in samples dehydrated at 400 °C [74]. Shang et al. studied the adsorption and separation performance of CH_4_ on three CHA-type molecular sieves with different pore diameters: Chabazite-K, SAPO-34, and SSZ-13. Experimental results showed that Chabazite-K had the highest selectivity (5.5). SSZ-13 had the largest pore volume and specific surface area but the lowest selectivity (2.5) and the highest CH_4_ adsorption capacity, reaching 1.38 mmol/g. With similar Si/Al ratios, the framework’s extra-framework metal cations determined the material’s adsorption capacity and selectivity. Due to the presence of metal cations, the pore size was narrowed, reducing CH_4_ adsorption capacity, while the increased polarization of CH_4_ enhanced the interaction between the pores and CH_4_. SSZ-13, with a larger pore volume, is suitable for recovering CH_4_ from low-concentration CH_4_ (CH_4_ < 20%). Reducing the crystal size of zeolites to achieve enhanced gas adsorption and separation performance has largely been unexplored and underestimated [67]. Compared to micron-sized zeolites, nanoscale zeolites have more adsorption sites and shorter diffusion paths. Yang and his team were the first to successfully prepare nano ZK-5 using β-cyclodextrin as a modulator. The crystal size of ZK-5 was reduced from micron-sized (3 μm) to nanoscale (50–100 nm), and nano ZK-5 exhibited superior specific surface area (370 m^2^/g) and pore volume (0.22 cm^3^/g) compared to the micron-sized samples. Compared to micron ZK-5, nano ZK-5 showed a 64% increase in CH_4_ adsorption capacity. At 298 K, nano ZK-5 showed a CH_4_ adsorption capacity of 1.34 mmol/g on commercial zeolites [68].

**Figure 7:**
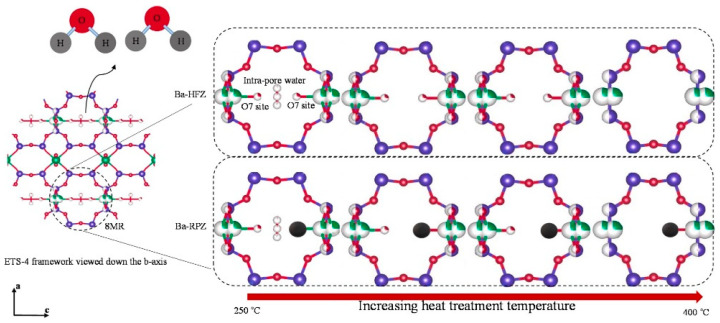
Schematic of Ba-ETS-4 structural changes during heat treatment process. Titanium atoms are presented in green. Silicon atoms are presented in blue. Oxygen and chlorine are presented in red and black, respectively [70].

Ion exchange modification of zeolites has also been a popular method for improving the separation of CH_4_/N_2_ mixtures in conventional zeolites over the past few decades. As early as 2004, Jayaraman et al. studied the adsorption performance of pure magnesium clinoptilolite for CH_4_/N_2_ separation and measured its high-pressure adsorption isotherms. They simulated pressure swing adsorption for pure clinoptilolite, magnesium-based clinoptilolite, and commercial adsorbent ETS-4. The purified clinoptilolite showed a slightly higher recovery rate than ETS-4 but a lower yield, with similar product purity (>95%). Their team also prepared mixed ion-exchange clinoptilolite with Mg^2+^/Ca^2+^, K^+^/Na^+^ and Mg^2+^/Na^+^ and studied the CH_4_/N_2_ separation performance of these ion-exchanged clinoptilolites. The results showed that Mg/Na (50/50) mixed ion-exchanged clinoptilolite exhibited good equilibrium and kinetic selectivity at low pressure, superior to pure clinoptilolite [75]. Subsequently, many researchers joined this field of study. Sethia et al. used volumetric gas adsorption to study zeolite-X exchanged with Mg^2+^, Ca^2+^, Sr^2+^, and Ba^2+^. The results showed that zeolite-X exchanged with Mg^2+^, Ca^2+^, Sr^2+^, and Ba^2+^ exhibited increased adsorption capacities for CH_4_ and N_2_. At 303 K and 1 bar, Sr^2+^-exchanged zeolite-X showed a CO adsorption capacity of 28.4 molecules per unit cell, while Ca^2+^-exchanged zeolite-X showed CH_4_ and N_2_ adsorption capacities of 18.8 and 13.8 molecules per unit cell, respectively. Ba^2+^-exchanged zeolite-X showed a CH_4_/N_2_ selectivity of 1.78 [76]. D.A. Kennedy et al. performed cation exchange modification of natural clinoptilolite using alkali metal ions, alkaline earth metal ions, transition metal ions, and acid treatment. They evaluated the composition and structural properties of the modified samples using EDS and XRD analysis and compared them with the original clinoptilolite samples. The results showed that cation-exchanged clinoptilolites exhibited a wide range of adsorption characteristics, making them suitable for various gas separation applications via pressure swing adsorption. The high adsorption selectivity of Cs^+^-exchanged clinoptilolite for CH_4_ favored CH_4_/N_2_ equilibrium separation. Due to pore blocking in Ca^2+^-exchanged clinoptilolite, CH_4_ equilibrium capacity and N_2_/CH_4_ selectivity were reduced. However, the potential of this material was limited due to increased micropore diffusion resistance. Although Li^+^- and Ni^2+^-exchanged clinoptilolites exhibited low CH_4_/N_2_ ideal selectivity, they showed high N_2_/CH_4_ kinetic selectivity, making them suitable for potential N_2_/CH_4_ kinetic separation [77]. Hao et al. ground, concentrated by weight, and treated clinoptilolite with ion exchange using different salt solutions. The modified clinoptilolite powders were then pelletized and used as adsorbents. Under conditions of 0.2 MPa and 298 K, the adsorbents were used for N_2_/CH_4_ separation in pressure swing adsorption (PSA). The results showed that the composites had micropores and a large number of mesopores, mainly formed by slit pores from layered stacking. NH_4_-Cp, Cs-Cp, and Cu-Cp adsorbents showed good equilibrium selectivity for CH_4_, with selectivities of 2.56, 2.31, and 1.95, respectively. Na-Cp had an N_2_/CH_4_ selectivity of 7.25, showing good equilibrium selectivity. Na-Cp adsorbent was suitable for CH_4_/N_2_ mixed gas separation, increasing CH_4_ concentration from 19.7% to 30.72% [78]. In 2019, Dean A. Kennedy et al. studied cation-exchanged clinoptilolite, obtaining modified clinoptilolite through cation exchange modification of natural clinoptilolite. At temperatures between 15 °C and 30 °C, Cs^3+^- and Fe^3+^-exchanged clinoptilolite had CH_4_/N_2_ selectivities ranging from 2.7 to 5.3 and 3.9 to 7.3, respectively, while typical activated carbon selectivities ranged from 2.1 to 5.5 [79]. Wu et al. developed amine ion-exchanged Y-type molecular sieves for CH_4_/N_2_ separation. Through simple ion exchange with tetramethylammonium cation (TMA+) and choline cation (Ch^+^), the resulting adsorbents significantly increased CH_4_ adsorption and decreased N_2_ adsorption. Compared to the original NaY, the CH_4_/N_2_ separation performance of the resulting samples greatly improved. At 25 °C and 100 kPa, the CH_4_/N_2_ selectivities of TMAY and ChY reached 6.32 and 6.50, respectively [69]. Mahsa Vosoughi et al. synthesized Na-ETS-4 using both Cl-containing and halogen-free methods, and the synthesized adsorbents (Ba-RPZ and Ba-HFZ) were ion-exchanged with Ba^2+^, as shown in Figure 7. They studied the adsorption equilibrium and kinetics of N_2_ and CH_4_ on the two Ba-ETS-4 adsorbents. Static volumetric adsorption was used to determine adsorption data at 30 °C and pressures from 0 to 100 kPa. The results showed that due to the gradual structural contraction with increasing heat treatment temperature, equilibrium capacity and adsorption rates gradually decreased. When the activation temperature increased to 400 °C, equilibrium selectivity and kinetic selectivity decreased due to additional structural deformation. The presence of Cl- in Ba-RPZ added extra steric hindrance, reducing adsorption capacity and gas diffusion coefficients [70].

**Figure 8:**
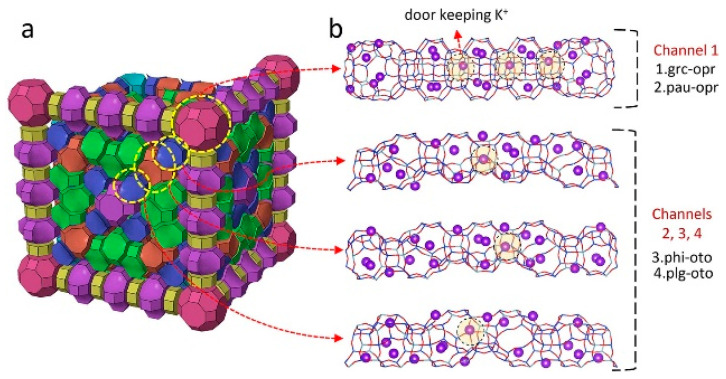
N_2_ and CH_4_ diffusion passes in ZSM-25: (**a**) 3D view of the ZSM-25 unit cell and (**b**) four unique channels connected through eight-membered rings as the main routes for gas diffusion consisting of four double-connected cages, namely, (1) grc-opr, (2) pau-opr, (3) phi-oto, and (4) plg-oto. The door-keeping cations are highlighted [82].

In 2023, Mousavi et al. used GCMC simulations to evaluate the CH_4_ and N_2_ adsorption capacities and selectivities of 1425 different alkali metal-exchanged zeolites, discovering that some specific zeolite frameworks could achieve equilibrium selectivity for N_2_. They also studied the effects of alkali metal types (Li^+^, Na^+^, K^+^, Rb^+^, and Cs^+^) and Si/Al ratios on the performance of each framework. The results showed that K^+^ cations exhibited the highest affinity for N_2_ adsorption, while the smaller Li^+^ cations had the highest gas absorption. Additionally, a lower Si/Al ratio favored N_2_/CH_4_ selectivity [80]. Furthermore, the development of ionic liquid zeolites has provided new ideas for improving the separation of CH_4_/N_2_ by zeolites. In 2022, Hu et al. studied the separation of CH_4_ from N_2_ using 100 kg of ionic liquid zeolite (ILZ) material in a pressure swing adsorption process. The CH_4_ concentrations in the feed gas were increased from 5.0% and 16.1% to 11.5% and 34.6%, respectively, with CH_4_ recovery rates higher than 80% [81]. Researchers have also conducted studies on composite modification of zeolite molecular sieves. In 2020, Tang et al. first synthesized nm/mm-sized (500 nm) K-KFI (Si/Al = 1/4.59) adsorbents using a hydrothermal method and ultrasound-assisted method, achieving a CH_4_ adsorption capacity of 1.05 mmol/g. The team also treated the zeolite with ultrasound and found that the ultrasound time significantly affected the surface of the K-KFI molecular sieve, reducing its particle size from 1.5 μm to 500 nm, confirming that the ultrasound-assisted method is a fast and effective way to synthesize nm/mm-sized zeolite molecular sieves [71]. In 2021, Zhao et al. reported a new K-ZSM-25 trapdoor molecular sieve material, as shown in Figure 8, where K^+^ acts as the “gatekeeper” cation. The temperature-dependent oscillation degree of K^+^ cations regulates the accessibility of the cages, controlling the material’s adsorption capacity. Experimental and theoretical results showed that the small-pore zeolite ZSM-25 provided a unique opportunity for effective N_2_ adsorption. This material exhibited good N_2_ capacity, excellent N_2_/CH_4_ selectivity (up to 34), outstanding kinetic effects, and regeneration potential at room temperature, making it well-suited for PSA-based industrial separation. The design of this trapdoor material opens new avenues for N_2_/CH_4_ separation in various fields, aiming to develop cleaner and more energy-efficient gas processing technologies. Additionally, adjustable pore accessibility offers potential applications for gas storage and molecular encapsulation [82].

**Figure 9:**
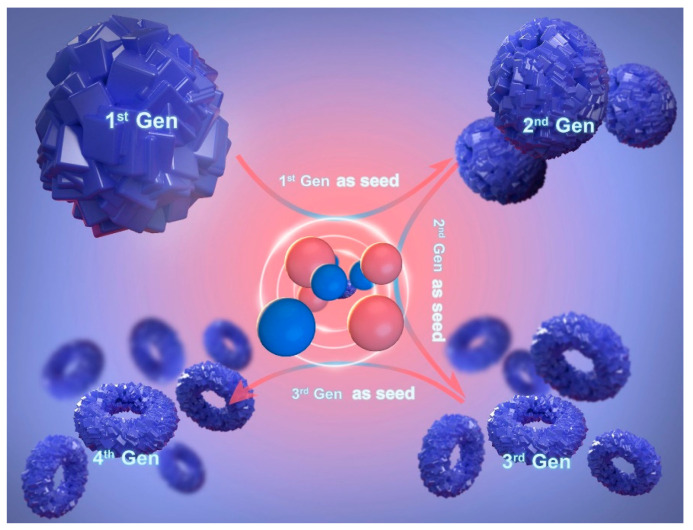
Synthesis of nanosized K-Chabazite by the seed-passaging route [72].

In 2021, Yang and colleagues reported a simple and green seed proliferation method to prepare ring-shaped hierarchical K-chabazite molecular sieve nanoclusters with macro-, meso-, and micro-porous structures, as shown in Figure 9. This continuous seed induction method does not require an organic template. By utilizing this unique nanotechnology, the resulting material demonstrated significantly increased CH_4_ adsorption capacity, gas diffusion rate, and separation productivity compared to commercially available adsorbents. Notably, the raw materials for this adsorbent are easily obtainable, and the synthesis route is environmentally friendly, offering the potential for industrial-scale production [72].

Recently, in 2024, Ghasemi and colleagues achieved a significant breakthrough by innovatively applying RHO-type zeolite membranes in the field of CH_4_/N_2_ separation. They demonstrated that N_2_ gas molecules can easily pass through the membrane channels, while CH_4_ molecules, due to their larger molecular diameter, cannot enter the membrane cavities. Their team conducted simulation studies on the N_2_ separation performance of RHO molecular sieve membranes for CH_4_/N_2_ mixtures at 298 K and a pressure difference of up to 30 MPa. The results showed that the RHO molecular sieve membrane exhibited very high permeability and selectivity for N_2_, surpassing the upper limit defined by Robeson, with a maximum permeability of 2.14 × 10^5^ GPU (gas permeation units). Additionally, they studied the effects of varying the feed gas composition and membrane thickness. The results indicated that permeability increased with decreasing membrane thickness, and the feed gas composition had a significant impact on the separation of CH_4_ and N_2_ [83].

### 3.3. Metal/Organic Frameworks (MOFs)

Metal/organic frameworks (MOFs), also known as porous coordination polymers (PCPs), are a new type of porous material formed by the complexation of inorganic metal centers (metal ions or metal clusters) with organic ligands. Due to their ultra-high specific surface area, ordered pore structure, adjustable pore size, and easily functionalizable framework surface, these materials have been widely applied in gas adsorption and storage, catalysis, bioimaging and sensing, drug delivery, magnetic devices, and nonlinear optics [84]. Recent research has significantly expanded the variety of these materials, mainly including the ZIF series [85], MIL series [86], and UIO series, among others, as shown in Table 5.

Researchers first conducted extensive research based on the difference in polarizability between CH_4_ and N_2_, utilizing the difference in equilibrium adsorption capacity exhibited by gases in metal/organic frameworks. In 2012, Möllmer and colleagues found that the CH_4_/N_2_ separation factors for [Cu(Me-4py-trz-ia)] and Basolite^®^ A100 were as high as 4.5 and 5.0, respectively. At 0.1 MPa and 298 K, they measured the CH_4_ adsorption capacities of Basolite A100 and [Cu(Me-4py-trz-ia)] to be 0.71 mmol/g and 1.12 mmol/g, respectively. This study was the first to demonstrate the excellent performance of metal/organic frameworks (MOFs) in the adsorption separation of CH_4_/N_2_ [88].

**Figure 10:**
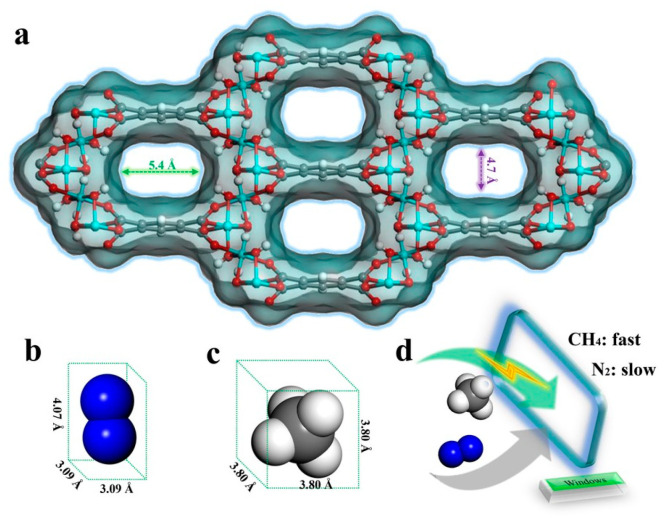
(**a**) 3D framework of MIL-120Al; (**b**,**c**) molecular size of CH_4_ and N_2_; (**d**) illustration of the different kinetic effects of CH_4_ and N_2_ through the window of MIL-120Al. Color code: C, gray; H, white; O, red; Al, cyan; N, blue [91].

The special microstructural environment within MOFs plays a crucial role in enhancing CH_4_ adsorption capacity. Li and colleagues reported a novel Cd-1,3,6,8-tetrakis(para-benzoic acid) pyrene framework, ROD-8, which possesses two types of one-dimensional structures (0.65 nm × 1.18 nm and 0.85 × 0.95 nm). The shortest distance between two parallel pyrene core planes in the structure is 0.435 nm. Under conditions of 298 K and 1 bar, the CH_4_/N_2_ separation factor reached 9.0, and the CH_4_ adsorption capacity was 0.77 mmol/g [89]. Liu and colleagues comprehensively characterized MOFs MMA-BPY (M = Co and Ni) and found that they exhibit good framework flexibility. Additionally, M-MA-BPY MOFs showed excellent stability in water and humid air. At 298 K and 1 bar, they demonstrated good CH_4_ capacities (0.92 and 1.01 mmol/g for Co- and Ni-MA-BPY, respectively) and significant CH_4_/N_2_ selectivity (CH_4_/N_2_ separation selectivity of 7.2 and 7.4 for Co- and Ni-MA-BPY, respectively) [90]. Subsequently, Feng and colleagues used suspension polymerization and in situ growth techniques to transform the morphology of Al-CDC from three-dimensional (3D) crystals to two-dimensional (2D) nanosheets, forming nanomembranes with higher adsorption efficiency and lower diffusion barriers. Experimental results showed that compared to bulk Al-CDC, its adsorption efficiency increased by 1.73 times, with a separation factor for CH_4_/N_2_ (50/50, *v*/*v*) mixture of 13.75 and a CH_4_ adsorption capacity of 1.32 mmol/g [113]. Zhang and colleagues studied an ultramicroporous MOF with nonpolar pore walls, MIL-120Al, which is constructed from infinite rod-like structural units and BTEC ligands. It features a unique aluminum-based three-dimensional open framework with ultramicroporous walls formed by appropriately sized benzene rings, as shown in Figure 10. Benefiting from the kinetic synergistic separation effect, this MOF exhibited excellent separation performance for CH_4_/N_2_ mixtures under dynamic conditions, comparable to the previously reported Al-CDC adsorption capacity [91].

**Figure 11:**
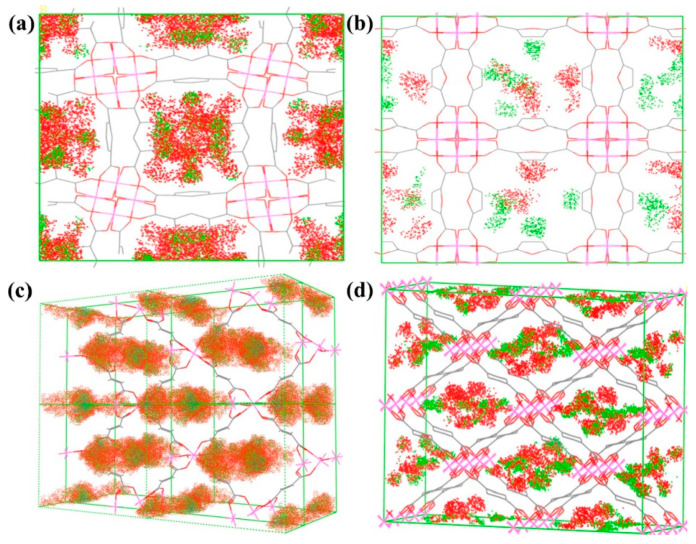
The simulated distribution of adsorption density on (**a**) CAU-10-H, (**b**) MIL-160, (**c**) Al-Fum, and (**d**) MIL-53(Al) during the adsorption process (red regions for CH_4_, green regions for N_2_) [92].

Huang and colleagues further explored the potential of Al-MOFs as CH_4_/N_2_ separation adsorbents by considering the effects of pore geometry and linker polarity. They synthesized two one-dimensional square Al-MOFs, 10-H and MIL-160, and two corresponding rhombic counterparts, Al-Fum and MIL-53(Al), using two bent ligands with different polarities and two linear ligands. Their research found that Al-Fum exhibited a CH_4_/N_2_ separation factor as high as 17.2 at 273 K and 1.0 bar. To verify the accuracy of the experimental results, they used Monte Carlo simulations to model the adsorption density distribution of CH_4_ and N_2_ in Al-MOFs, as shown in Figure 11. The results indicated that the adsorption density distribution of CH_4_ molecules in the channels of Al-MOFs was significantly higher than that of N_2_ molecules, consistent with the experimental results [92].

Considering characteristics such as biocompatibility, cost, toxicity, and natural abundance, Chang and colleagues utilized the properties of calcium metal to prepare a novel calcium-based metal/organic framework material, SBMOF-1 (ligand: 4,4′-SDB), for CH_4_/N_2_ adsorption separation. This Ca-MOF, containing low-polarity polyaromatic organic ligands, exhibited a CH_4_/N_2_ separation factor as high as 11.5, along with a relatively high CH_4_ adsorption capacity of approximately 0.92 mmol/g [93]. Additionally, research has found that pore size significantly impacts CH_4_/N_2_ adsorption separation. He and colleagues designed UTSA-30 (Ln-MOFs) with three-dimensional channels. By degassing acetone-exchanged UTSA-30 under high vacuum at room temperature, they produced UTSA-30a, which exhibited a CH_4_ adsorption capacity of 0.6 mmol/g and a CH_4_/N_2_ separation factor of up to 5.0 at 298 K and 1 bar [94]. Ma and colleagues successfully designed and synthesized a carboxyl-functionalized PAF material, PAF-26-COOH. Post-metallization of PAF-26-COOH produced a series of PAF-26-COOM derivatives (M = Li, Na, K, Mg). These functionalized materials are ultramicroporous, with DFT results showing pore sizes in the range of 4–6 Å. They exhibited good separation selectivity of 4.2–6.5 under conditions of 298 K and 110 kPa [95].

**Figure 12:**
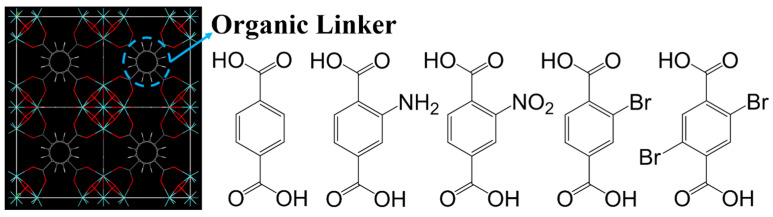
Crystallographic structure of UiO-66 unit cell and schematic of organic linkers of UiO-66-X materials [98].

Hu and colleagues used divalent metal ions to adjust the pore size and polarity of molecular sieves. The results showed that metal formates have different affinities for CH_4_, exhibiting different CH_4_ adsorption capacities and CH_4_/N_2_ selectivities in the order of Ni > Co > Mg > Mn. Among them, [Ni_3_(HCOO)_6_] demonstrated the highest CH_4_ adsorption capacity (0.81 mmol/g) and CH_4_/N_2_ selectivity (6.5) in dynamic adsorption experiments at 0.1 MPa and 298 K. This indicates an optimal synergy between pore contraction and surface properties among the [M_3_(HCOO)_6_] frameworks. The adsorption behavior of [M_3_(HCOO)_6_] was studied using NH_3_-TPD, revealing two different adsorption states of NH_3_ molecules within [M_3_(HCOO)_6_], one where gas molecules reside within the pores and another where gas molecules adsorb onto the coordinating metal ions or exposed oxygen-induced adsorption sites. This confirms the important role of metal ions in adjusting pore size and internal surface properties [96]. Building on previous work, Guo and colleagues synthesized [Ni_3_(HCOO)_6_] using a novel solvent-free method. At 298 K and 100 kPa, the CH_4_ adsorption capacity was 0.82 mmol/g (comparable to the previous value), and the CH_4_/N_2_ selectivity was 6.18. This method converts the nickel(II) precursor and formic acid into the [Ni_3_(HCOO)_6_] framework at mild temperatures, avoiding the use of harmful solvents and providing an environmentally friendly route [114]. Shi and colleagues studied the CH_4_/N_2_ adsorption separation performance of three SOD-type ZIF materials (ZIF-8, ZIF-90, and SIM-1 (ZIF-94)) and one RHO-type material (ZIF-93). The results showed that SIM-1 (ZIF-94) exhibited the highest CH_4_ adsorption capacity (1.51 mmol/g at 298 K and 1 bar). Analysis of the structures and organic ligands of the four ZIFs revealed that the narrow pore size (0.84 nm in SIM-1) plays a key role in CH_4_ adsorption. IAST calculations indicated that the CH_4_/N_2_ separation factor of SIM-1 (ZIF-94) is seven times higher than that of reported ZIFs and most porous materials. The CH_4_ retention time in SIM-1 is nearly ten times longer (20 min) than in the other three materials. However, SIM-1 cannot currently be scaled up cost-effectively, necessitating the development of greener synthesis methods in the future [97]. Kim and colleagues introduced functional groups (-H, -NH_2_, -NO_2_, -Br, and -Br_2_) into the pores of hydrothermally stable zirconium-based metal/organic frameworks (MOFs), as illustrated by the organic linkers in Figure 12. Experimental and molecular simulation results showed that UiO-66-Br_2_ exhibited significant adsorption performance, with a CH_4_ adsorption capacity of 0.72 mmol/g and a CH_4_/N_2_ selectivity of 5.06. Simulations and calculations confirmed that introducing bulky functional groups can exploit their high polarizability and reduce pore size, thereby enhancing CH_4_ adsorption capacity [98].

**Figure 13:**
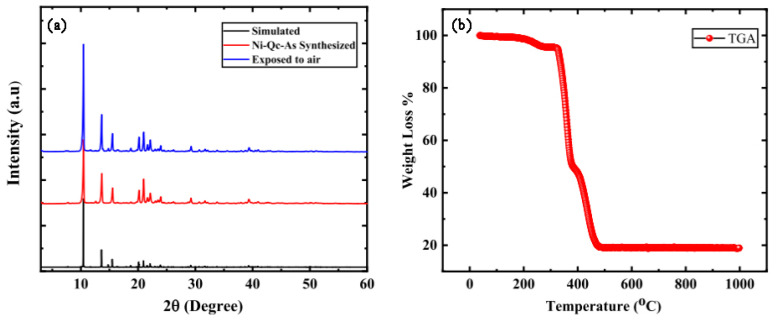
(**a**) XRD patterns of experimental and simulated Ni-Qc-5 MOF (**b**) Thermogravimetric analysis of Ni-Qc-5 MOF [100].

Wang and colleagues prepared four nickel-based diamond coordination frameworks (Ni(ina)_2_, Ni(3-ain)_2_, Ni(2-ain)_2_, Ni(pba)_2_) by introducing functional sites (-NH_2_) or altering the length of the ligands to fine-tune the pore chemistry and pore size, thereby achieving ultra-high CH_4_ adsorption and excellent separation performance. Among these, Ni(ina)_2_ exhibited significant differences in CH_4_ and N_2_ adsorption, with a CH_4_ adsorption capacity of 1.665 mmol/g and a CH_4_/N_2_ selectivity of 15.8 at 298 K and 100 kPa. Further GCMC simulations and DFT calculations were conducted to study the mechanisms of selective CH_4_ capture by Ni(ina)_2_ and Ni(3-ain)_2_. The results indicated that Ni(ina)_2_ and Ni(3-ain)_2_ provided suitable accommodation spaces for CH_4_ molecules. However, the ink-bottle-shaped channels with very narrow pore sizes in Ni(2-ain)_2_ were unsuitable for capturing CH_4_. Conversely, the larger pore sizes in Ni(pba)_2_ made it difficult to capture CH_4_ due to weaker intermolecular interactions [99]. Qadir and colleagues successfully synthesized a microporous Ni-Qc-5 using low-polarity, low-toxicity, and naturally abundant polyaromatic ligands, which exhibited excellent CH_4_ and N_2_ separation performance. At room temperature and atmospheric pressure, the CH_4_ adsorption capacity was 1.3 mmol/g, with a CH_4_/N_2_ selectivity of 7.0. The PXRD pattern of the sample obtained using a PANalytical X’pert diffractometer (Malvern, UK) matched well with the simulated pattern derived from crystallographic data, as shown in Figure 13. TGA results indicated that the Ni-Qc-5 MOF sample remained stable below 300 °C, with degradation occurring only above this temperature. Due to its stability and ease of regeneration, this adsorbent has promising applications for capturing coal mine methane at low pressures [100].

**Figure 14:**
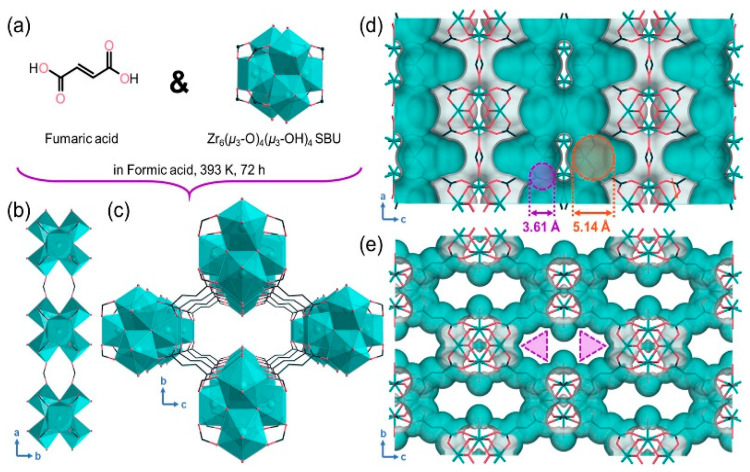
(**a**) Chemical structure of fumaric acid ligand and Zr_6_(μ3-O)_4_(μ3-OH)_4_ cluster in MIP-203-F. (**b**) Formate-linked Zr6-oxo cluster chain along the a-axis. (**c**) Framework structure of MIP-203-F with the hydroxyl group-divided dual triangular 1D pore. (**d**) Connolly surface of MIP-203-F with a probe radius of 1.82 A viewed along the b-axis. (**e**) Van der Waals surface of MIP-203-F viewed along the a-axis [103].

Chang and colleagues were the first to apply the ultramicroporous copper-dicyanoimidazole MOF (NKMOF-8-Me) for CH_4_/N_2_ separation. Due to the introduction of aromatic imidazole derivatives, this MOF features regular nonpolar/inert pore surfaces and appropriate pore sizes, resulting in a shorter average distance between CH_4_ and the pore walls, thereby enhancing CH_4_ adsorption affinity. The adsorption results indicated that this material exhibits the highest CH_4_ adsorption capacity (1.76 mmol/g) and a CH_4_/N_2_ adsorption selectivity of 9.0 (50:50 *v*/*v*). This MOF can be synthesized quickly and easily on a large scale at room temperature and demonstrates high structural stability under various conditions, including exposure to water vapor, boiling water, acid solutions (pH = 1), alkaline solutions (pH = 11), and high temperatures (700 K) [101]. Guo and colleagues reported a stable ultramicroporous Cu(I)-based metal/organic framework (MOF), NKMOF-8-Br, which exhibited an excellent CH_4_ adsorption capacity (1.84 mmol/g) and high CH_4_/N_2_ (50/50, *v*/*v*) selectivity of 8.9 at room temperature and atmospheric pressure. The CH_4_ adsorption capacity surpasses all water-stable MOFs and other types of adsorbents used for CH_4_/N_2_ separation [102]. Chen and colleagues reported a Zr-based metal/organic framework (MIP-203-F) featuring a rhombic one-dimensional (1D) dual-pore structure. The presence of side-chain -OH groups effectively divides the structure into two symmetric wall-sharing triangular pores, providing the material with optimal pore size and numerous synergistic polar sites. This promotes efficient CH_4_ adsorption, taking advantage of its high polarizability, and overcomes the trade-off between CH_4_ capacity and CH_4_/N_2_ selectivity. The resulting MIP-203-F framework structure with 1D channels (Figure 14c) has a pore limiting diameter (PLD) of 3.61 Å and a maximum cavity diameter (LCD) of 5.14 Å, as shown in Figure 14d,e [103].

However, Chang and colleagues achieved precise pore size tuning by altering the degassing temperature, inducing the dynamic switching of the initial two-dimensional (2D) framework to prepare Cu-MOF-SCH_3_. The empty framework and original state can be fully switchable under vacuum and upon exposure to water vapor, demonstrating excellent resistance to water vapor. Notably, this material can be produced on a large scale using supergravity technology, and Cu-MOF-SCH_3_ exhibits the highest STY (space–time yield) among all 2D MOFs [104]. Studies have shown that the special adsorption sites of metal/organic frameworks can further enhance the CH_4_/N_2_ adsorption separation performance. Li and colleagues investigated two isostructural MOFs, [Cu(1,3-BDC)(H_2_O)]·2H_2_O and Cu(1,3-BDC)(PY)_2_, and found that the introduction of the organic ligand pyridine in Cu(1,3-BDC)(PY)_2_ provided more CH_4_ adsorption sites, improving the CH_4_/N_2_ separation factor to 20.14 at 1 MPa [105]. Li and colleagues were the first to prepare an ultramicroporous [Co_3_(C_4_O_4_)_2_(OH)_2_] (C_4_O_4_^2−^ = squarate) with enhanced negatively charged oxygen binding sites, achieving a maximum CH_4_/N_2_ separation factor of 12.5. Due to its high thermal stability and low regeneration cost, this material shows great potential for industrial applications [106]. Niu and colleagues reported a metal framework, ATC-Cu, which has unique relatively adjacent open metal sites that provide very strong binding sites for CH_4_ at relatively low pressures. ATC-Cu can adsorb 2.90 mmol/g of CH_4_ at room temperature and atmospheric pressure, and the CH_4_/N_2_ selectivity of ATC-Cu, calculated from binary equimolar mixtures, is as high as 9.7 [107]. Lv and colleagues proposed enhancing the CH_4_ adsorption affinity by controlling the polar sites on the pore walls of aluminum-based metal/organic frameworks (MOFs). Their experiments showed that at 298 K and 1 bar, the CH_4_/N_2_ selectivity of CAU-21-BPDC (11.9) was significantly higher than that of CAU-8-BPDC (4.9). CAU-21-BPDC, with four highly symmetric polar sites, exhibited a CH_4_/N_2_ selectivity 2.4 times greater than CAU-8-BPDC, which lacks these polar sites. The CH_4_ adsorption capacity of CAU-8-BPDC was 0.85 mmol/g (298 K, 100 kPa), while that of CAU-21-BPDC was 0.99 mmol/g (298 K, 100 kPa). Additionally, CAU-21-BPDC showed better water stability compared to CAU-8-BPDC, attributed to its lower water vapor adsorption capacity, making it less likely for water vapor to replace the organic ligands in the CAU-21-BPDC framework and disrupt the metal/ligand bonds. This study first confirmed that, besides the strength of metal/ligand bonds, the coordination number of metal ions, the degree of framework interpenetration, and the hydrophobicity of the pore wall surfaces, water vapor adsorption capacity also affects the water stability of MOFs [108]. Zheng and colleagues synthesized a novel 2D layered metal/organic framework, Ni(4-DPDS)_2_CrO_4_ (4-DPDS = 4,4′-dipyridyldisulfide), for the first time. This novel 2D MOF exhibited excellent or even better high stability compared to previously reported 3D MOFs. At 273 K and 1 bar, it demonstrated a CH_4_ adsorption capacity of 0.95 mmol/g and a CH_4_/N_2_ selectivity of 7.3. Density functional theory calculations indicated that the energetically favorable binding sites for CH_4_ molecules were located in the middle of the cavities modified by CrO_4_^2−^ anions. The angled inorganic anions provide polar sites and bring the guest/host interactions very close, thereby enhancing the affinity of Ni(4-DPDS)_2_CrO_4_ for CH_4_ [109]. Considering the high cost and environmental issues of synthesizing metal/organic frameworks (MOFs), Fang and colleagues used fly ash as a raw material. By using sulfuric acid as an extractant and applying direct leaching and roasting processes, they prepared CFAx-FumMOF-y. The CH_4_/N_2_ selectivity ranged from 3.98 to 4.65, with CH_4_ adsorption capacities between 0.844 and 0.895 mmol/g. Among them, CFAs-FumMOF-1 exhibited the highest adsorption selectivity of 4.56. The specific surface area and pore volume of CFAx-FumMOF-y reached 1164.94–1073.08 m^2^/g and 0.40–0.36 cm^3^/g, respectively, which are comparable to those of Al-FumMOF (1287.55 m^2^/g and 0.42 cm^3^/g) [110]. Recently, Wang and colleagues addressed the challenge of maintaining the high selectivity of porous materials under high pressure by preparing a low-polarity microporous membrane Ni(TMBDC)(DABCO)_0.5_. Experimental results showed that it achieved a CH_4_ adsorption capacity of 4.23 mmol/g and a CH_4_/N_2_ separation factor of 5.1 at 298 K and 10 bar [111]. Liu and colleagues developed a titanium metal/organic framework adsorbent, ZSTU-1, with dual nano traps. These nano traps enhance CH_4_ adsorption capacity (1.37 mmol/g) through C-H···O hydrogen bonds and multiple C-H···π interactions, with CH_4_/N_2_ selectivity ranging from 21.6 to 12.0. Notably, in a single separation of an equimolar CH_4_/N_2_ mixture, the CH_4_ productivity reached an unprecedented 13.1 L/kg. Furthermore, in the presence of air or water vapor, it far surpassed the CH_4_ productivity of Co_3_(C_4_O_4_)_2_(OH)_2_ (0.29 L/kg) [112].

Researchers have conducted extensive studies on the CH_4_/N_2_ adsorption separation performance of MOF materials and have made significant progress. However, some MOFs still suffer from issues such as poor selectivity, low adsorption capacity, high synthesis costs, and poor water stability. These drawbacks greatly affect the adsorption performance and limit practical applications. Given the inherent advantages of MOFs, such as ultra-high specific surface area, adjustable pore size, and easily functionalizable framework surfaces, the continuous search for novel MOF materials with excellent performance is of great importance to meet the demands of large-scale industrial applications in the near future.

## 4. Conclusions and Perspectives

Developing new technologies for the purification of low-concentration CH_4_ and enriching unconventional natural gas not only alleviates the shortage of natural gas but also significantly reduces the greenhouse effect and environmental pollution. The main bottleneck in CH_4_ enrichment is the removal of nitrogen from low-concentration gas. Adsorption separation, as an effective CH_4_/N_2_ separation technology, has advantages such as low cost and simple operation, making it commercially promising. The development of high-performance adsorbents is key to continuously improving nitrogen removal efficiency. Research on carbon-based materials, zeolite molecular sieves, and metal/organic frameworks (MOFs) adsorbents for CH_4_/N_2_ adsorption has been extensive, with a good understanding of the adsorption behaviors of various adsorbents and the principles for performance optimization. However, there are still areas that need improvement, requiring the exploration of new strategies to enhance the attraction differences between methane and nitrogen with the adsorbents. For example, the surface properties and pore optimization of carbon-based materials, as well as the controllability and reproducibility of heteroatom-doped carbon-based adsorbents, deserve attention and further research. The issue of high surface polarization and diffusion kinetics mutual inhibition in zeolite molecular sieves and how to use metal ions to control the surface potential for directional adjustment, particularly in finely tuning the pore size and surface properties of ultramicropores (<8 μm) at the molecular scale, are also important. MOFs, which combine the advantages of the former two, exhibit unique benefits in CH_4_/N_2_ adsorption separation. However, challenges remain in adsorption selectivity, adsorption capacity, operational stability, and commercial application value. Further research and optimization are needed to enhance water stability and control synthesis costs. There is significant room for development in structural modification techniques to purify complex gas mixtures (isomers, gas isotopes). In the future, coupling multiple technical principles or introducing high-tech methods such as 3D printing (DIW) will be directions for researchers to explore continuously.

This paper does not provide any theoretical, equation, or calculation background; it mainly focuses on processes, materials, and properties. The theoretical background and main equations can be found in the following references [115,116,117].

## Figures and Tables

**Table 1:** Physical and Chemical Properties of CH_4_ and N_2_ [15].

Molecular	σ/nm	α/nm^3^	μ/(C·m)	Θ/(C·m^2^)	Tc/K
CH_4_	0.380	2.448 × 10^−3^	0	0.067 × 10^−40^	190
N_2_	0.364	1.710 × 10^−3^	0	5.134 × 10^−40^	126

σ—kinetic diameter; α—polarizability; μ—dipole moment; Θ—quadrupole moment; Tc—critical temperature.

**Table 2:** Principles and Technical Analysis of Low-Grade Gas Purification.

Purification Technology	Principle	Strengths and Weaknesses
Cryogenic distillation technology [16]	Utilizes the difference in boiling points between CH_4_ and N_2_ to separate the mixed gases after liquefaction.	The product gas has a high purity and recovery rate of CH_4_, but the operation conditions are demanding, the equipment investment is substantial, and the risk factor is high.
Membrane separation technology [17]	Utilizes the difference in the permeation rates of CH_4_ and N_2_ through the membrane, driven by pressure, through steps of dissolution, diffusion, and desorption to achieve purification.	High separation efficiency, low energy consumption, simple operation, and sustainable operation, but low membrane selectivity, high cost, and poor mechanical properties.
Chemical absorption technology [18]	Utilizes specific chemical absorbents to react with impurities in the CH_4_ gas, thereby achieving CH_4_ purification.	Good purification effect, low operating pressure, minimal CH_4_ loss, but high energy consumption for processing, complex regeneration process, and high investment cost.
Hydrate technology [19]	Utilizes the preferential encapsulation of CH_4_ over N_2_ in hydrate cavities when gas and water form hydrates under low temperature and high-pressure conditions, thereby achieving the separation of mixed gases.	Safe technology, highly efficient and energy-saving, with low pressure loss and short industrial trial process, but with low separation efficiency and relatively low product purity.
Adsorption separation technology [20,21]	Utilizes the high selectivity of solid adsorbents for CH_4_, leveraging the significant differences in gas adsorption capacities to achieve efficient CH_4_ purification.	Mature technology, low energy consumption, simple process, flexible operation, but high performance requirements for adsorbents.

**Table 3:** Adsorption Performance of Carbon-based Materials for CH_4/_N_2_.

AC	CH_4_/N_2_ Selectivity	CH_4_ Adsorption Capacity (mmol/g)	Reference
GAC(C-12)	3.17 ^d^	2.3 ^d^	[32]
C-12	4.8 ^e^	13.0 ^e^	[33]
AC-1-400	3.38 ^f^	0.87 ^f^	[34]
ClCTF-1-650	8.1 ^b^	1.47 ^b^	[35]
N-WAPC	7.62 ^b^	1.01 ^b^	[36]
OTSS-2-450	4.9 ^b^	0.85 ^b^	[37]
ACK_2_N_1_	7.11 ^a^	3.0 ^a^	[38]
AC NH_3_·H_2_O-10%	4.62 ^b^	1.1 ^b^	[39]
KCl/AC	5.33 ^b^	2.849 ^b^	[40]
SCs	5.7 ^b^	1.86 ^b^	[41]
C-PVDC 700	14.7 ^b^	1.57 ^b^	[42]
CMS-G	4.74 ^c^	1.41 ^c^	[43]
CMS-P-N	3.32 ^b^	0.95 ^b^	[44]
PCF-Co-0.5	0.97 ^b^	6.8 ^b^	[45]

^a^ 273 K, 1 bar; ^b^ 298 K, 1 bar; ^c^ 1023 K, 1 bar; ^d^ 298 K, 1 MPa; ^e^ 298 K, 6 MPa; ^f^ 293 K, 1 bar.

**Table 4:** Adsorption Performance of Zeolites for CH_4_/N_2_.

Zeolites	CH_4_/N_2_ Selectivity	CH_4_ Adsorption Capacity (mmol/g)	Reference
5A	2.5 ^b^	0.71 ^a^	[66]
13X	1.23 ^b^	0.5 ^a^	[66]
silicalite-1	3.92 ^b^	0.652 ^a^	[66]
Beta	2.58 ^b^	0.558 ^a^	[66]
Chabazite-K	5.5 ^a^	0.7 ^a^	[67]
SAPO-34	3.1 ^a^	0.73 ^a^	[67]
SSZ-13	2.7 ^a^	1.38 ^a^	[67]
Nano-ZK-5	4.4 ^a^	1.3 ^a^	[68]
TMAY	6.32 ^a^	0.52 ^a^	[69]
ChY	6.5 ^a^	0.41 ^a^	[69]
Na-ETS-4	2.65 ^c^	0.44	[70]
HT-K-KFI	4.6 ^a^	0.83 ^a^	[71]
K-Chabazite	5.5 ^a^	0.70 ^a^	[72]

^a^ 298 K, 1 bar; ^b^ 298 K, 1 bar (V(CH_4_):(N_2_) = 1:1 Breakthrough Curve); ^c^ 303 K, 1 bar.

**Table 5:** Adsorption Performance of Metal/Organic Frameworks (MOFs) for CH_4_/N_2_.

MOF	CH_4_/N_2_ Adsorptive Selectivity	CH_4_ Adsorption Capacity (mmol/g)^−1^	Reference
MOF-5	1.13 ^d^	0.13 ^b^	[87]
MOF-177	4.00 ^d^	0.59 ^b^	[87]
[Cu(Me-4py-trz-ia)]	4.5 ^b^	0.71 ^b^	[88]
Basolite A100	5.0 ^b^	1.12 ^b^	[88]
ROD-8	9.0 ^b^	0.77 ^b^	[89]
Co-MA-BPY	7.2 ^b^	0.92 ^b^	[90]
AL-CDC@PA	13.75 ^d^	1.32 ^b^	[90]
MIL-120Al	6.0 ^b^	1.3 ^b^	[91]
AL-Fum	17.2 ^a^	1.14 ^c^	[92]
MIL-53(AL)	6.8 ^a^	0.57 ^a^	[92]
CAU-10-H	7.0 ^a^	0.74 ^a^	[92]
MIL-160	8.8 ^a^	0.47 ^c^	[92]
SBMOF-1	11.5 ^b^	0.92 ^b^	[93]
UTSA-30	5.0 ^b^	0.60 ^b^	[94]
PAF-26-COOH	4.2 ^b^	0.54 ^b^	[95]
Ni_3_(HCOO)_6_	6.5 ^b^	0.81 ^b^	[96]
ZIF-94	7.0 ^b^	1.51 ^b^	[97]
UiO-66-Br_2_	5.06 ^b^	0.72 ^b^	[98]
Ni(ina)_2_	15.8 ^b^	1.67 ^b^	[99]
Ni-Qc-5	7.0 ^b^	1.30 ^b^	[100]
NKMOF-8-Me	9.0 ^d^	1.76 ^b^	[101]
NKMOF-8-Br	8.9 ^d^	1.84 ^b^	[102]
MIP-203-F	8.9 ^b^	1.16 ^b^	[103]
Cu-MOF-SCH_3_	15.0 ^b^	0.67 ^b^	[104]
[Cu(1,3-BDC)(H_2_O)]·2H_2_O	2.1 ^d^	0.34 ^b^	[105]
Cu(1,3-BDC)(PY)2	20.1 ^d^	0.72 ^b^	[105]
[Co_3_(C_4_O_4_)_2_(OH)_2_]	12.5 ^b^	0.88 ^b^	[106]
ATC-Cu	9.7 ^b^	2.90 ^b^	[107]
CAU-21-BPDC	11.9 ^b^	0.99 ^b^	[108]
CAU-8-BPDC	4.9 ^b^	0.85 ^b^	[108]
Ni(4-DPDS)_2_CrO_4_	7.3 ^a^	0.95 ^a^	[109]
CFAs-FumMOF-1	4.56 ^b^	0.844–0.895 ^b^	[110]
Ni(TMBDC)(DABCO)_0.5_	5.1 ^e^	4.23 ^e^	[111]
ZSTU-1	12–21.6 ^b^	1.37 ^b^	[112]

^a^ 273 K, 1 bar; ^b^ 298 K, 1 bar; ^c^ 273 K, 1 bar (CH_4_:N_2_ = 1:1); ^d^ 298 K, 1 bar V(CH_4_):(N_2_) = 1:1 Breakthrough Curve); ^e^ 298 K, 10 bar.
